# Steatotic Liver Disease Education Enhances Knowledge and Confidence to Adhere to Provider Recommendations in Diverse and Vulnerable Populations

**DOI:** 10.1016/j.gastha.2024.11.005

**Published:** 2024-11-20

**Authors:** Shyam Patel, Diana Partida, Catherine Magee, Flor E. Garza Romero, Jennifer Y. Chen, Michelle Tana, Mandana Khalili

**Affiliations:** 1 Division of Gastroenterology and Hepatology, Department of Medicine, University of California San Francisco, San Francisco, California; 2 Division of Gastroenterology and Hepatology, Department of Medicine, Zuckerberg San Francisco General Hospital, San Francisco, California; 3 Department of Medicine, California Pacific Medical Center, San Francisco, California

**Keywords:** Beliefs, Alcohol-Associated Liver Disease, Underserved Populations, Health Disparity, Metabolic Dysfunction-Associated Steatohepatitis

## Abstract

**Background and Aims:**

Patient knowledge of steatotic liver disease (SLD) is suboptimal. We assessed the impact of SLD education on patient knowledge and confidence to follow provider recommendations among a diverse vulnerable population.

**Methods:**

In this prospective study from February 19, 2020, to January 31, 2024, 296 adults with SLD were surveyed before and after receipt of formal SLD education. Linear regression (adjusted for age, sex, race) assessed factors associated with baseline SLD knowledge score and its change after education (delta in prescores and postscores), along with confidence to follow provider recommendations following receipt of education.

**Results:**

Participant characteristics were as follows: median age 53 years, 40.9% male, 55.1% Hispanic (27.0% Asian and 10.5% White), and 23.8% reported heavy alcohol use. SLD knowledge and confidence to follow provider recommendations increased posteducation (all *P* < .05). On multivariable analyses, greater than high school education (vs high school or less) (coef. 0.62), perceived severity of disease (coef. 0.62), treatment efficacy (coef. 1.38), self-efficacy to discuss SLD (coef. 0.71), and perceived susceptibility to disease risk (coef. 0.93) were associated with greater baseline knowledge (all *P* < .05). Following education, heavy alcohol use (vs none) was associated with greater change in knowledge (coef. 0.74), while perceived severity (coef. −0.52) and treatment efficacy (coef. −0.72) were associated with lesser change in knowledge (all *P* < .05). While perceived barriers (coef. −0.14) were associated with less confidence, self-efficacy to discuss SLD, older age, Hispanic, and other race was associated with greater confidence to follow provider recommendations (coef. 0.38, 0.18, 0.64, and 1.26, respectively, all *P* < .05).

**Conclusion:**

Formal SLD education enhanced knowledge and confidence to follow provider recommendations in Hispanics and heavy alcohol users. SLD education is integral to SLD management in safety net populations.

## Introduction

Steatotic liver disease (SLD), including metabolic dysfunction–associated SLD (MASLD) and alcohol-associated liver disease affects nearly 1 in 3 individuals in the US.^1^ Moreover, SLD disproportionately impacts vulnerable populations, such as those who are socioeconomically disadvantaged and racial minority groups.^2^ Despite a growing burden of disease in already at-risk communities, knowledge and awareness of SLD on a patient level, even after a diagnosis has been established, has historically been low.^3^, ^4^, ^5^ Furthermore, health-care providers have reported discomfort and lack of knowledge in discussing SLD with affected patients.^6^ Knowledge of SLD is important for care and management, especially since the mainstay of treatment is lifestyle modification (eg, healthy eating, weight loss, physical activity, alcohol reduction or cessation).^7^^,^^8^

Health education programs have the potential to facilitate the uptake of knowledge and promote adherence to provider recommendations in individuals affected by SLD. Prior studies within the viral hepatitis literature describe the impact of formal education in safety net hospital systems, reinforcing that disease knowledge influences health behaviors, and importantly uptake of health-care recommendations in this population.^9^, ^10^, ^11^, ^12^ However, few studies to date have focused on assessing knowledge, attitudes, and barriers regarding SLD care, nor have assessed the impact of an educational program on these measures in vulnerable and racially diverse populations.

As the drivers of SLD are behavioral, societal, and genetic in nature, a multidimensional model should be applied when implementing and designing interventions for SLD.^13^ Here, we use the Health Behavior Framework, a theoretical model providing useful tools for guiding the complex interplay of factors that influence SLD, which has previously been used and validated for community-level interventions and models of care in chronic liver disease.^11^^,^^12^^,^^14^, ^15^, ^16^ In this study, we aim to assess factors associated with baseline knowledge and the impact of a formal SLD educational program on change in knowledge and confidence to adhere to provider recommendations in a racially diverse and vulnerable population with SLD.

## Methods

### Study Population

From February 19, 2020, to January 31, 2024, the study recruited adult patients (≥18 years old) receiving care at hepatology clinics within the San Francisco safety net system. Participants with a diagnosis of SLD, defined as presence of steatosis on liver biopsy or imaging (eg, liver ultrasound, MRI/CT abdomen and pelvis) and documentation by a liver specialist from either metabolic-dysfunction and/or alcohol-associated etiologies were included. All eligible participants with a hepatology appointment were approached either via phone call or in-person at or after their visit. Patients with psychiatric or medical comorbidities preventing participation in the study or those unable to provide consent were excluded. The study was approved by the Institutional Review Board of the University of California, San Francisco and Zuckerberg San Francisco General Hospital.

### Study Intervention

Enrolled participants completed a 1-hour education session (in-person or remotely via Zoom) by a designated hepatology nurse practitioner using a standardized PowerPoint slide format. There was a maximum of 10 participants per education session. The content of our education session consisted of information regarding SLD natural history, prevention of disease progression, and management, including lifestyle modification recommendations (eg, healthy diet, weight loss, exercise, and alcohol reduction/cessation). The education session was conducted in-language with the help of certified medical interpreters for non-English speakers as needed. Moreover, the education materials were also translated to Spanish and Chinese (the most common non-English languages of the clinic population). Preeducation and posteducation questionnaires were completed by each participant. Our study questionnaire included sociodemographic information, as well as questions regarding SLD knowledge, beliefs about SLD, barriers to SLD care, and confidence to adhere to provider recommendations regarding SLD care. Participants received a $25 gift card after study completion.

### Questionnaire Design and Data Collection

The questionnaire instrument was developed using the Health Behavior Framework with input from expert hepatologists and behavioral scientists experienced in health behavior change research and information from published studies in patients with liver disease.^10^^,^^11^^,^^14^, ^15^, ^16^, ^17^, ^18^, ^19^ Additional items were added from previously validated questionnaires related to alcohol use, medical mistrust of providers, medical research, and disparities in care.^18^, ^19^, ^20^ The survey instrument was developed in English and translated into Spanish and Cantonese and further languages were verbally translated by a verified translating service (Table A1). The questionnaire was developed and implemented before the change in nomenclature in favor of SLD.^21^

Electronic medical records were used to collect clinical history, laboratory data, and relevant imaging findings. Using the National Institute of Alcohol Abuse and Alcoholism questionnaire, alcohol use in the past 12 months was grouped into 3 categories: none, moderate (≤1 drink/day for women, and ≤2 drinks/day for men), and heavy (>moderate).^20^ Body mass index information was categorized and race-adjusted as follows: normal <25 kg/m^2^ (<23 kg/m^2^ if Asian/Pacific Islander [API]), overweight 25–29.9 kg/m^2^ (23–27.4 kg/m^2^ if API), and obese ≥30 kg/m^2^ (≥27.5 kg/m^2^ if API).^22^

Following sociodemographic and clinical measures, the survey questionnaire items were organized into 5 domains: (1) SLD knowledge; (2) beliefs about SLD; (3) barriers to seeking care or managing their SLD (referred to as “barriers to SLD care” throughout); (4) medical mistrust; and (5) confidence to adhere to provider recommendations for SLD care.

### Statistical Analyses

Composite scores for each of the domains and subdomains were calculated from responses to questions designed to assess these factors as follows: (1) “SLD knowledge” score was computed as the number of correct responses to 8 questions (1 for correct, 0 for incorrect or do not know; max score. 10); (2) scores for each “beliefs about SLD” subdomain were determined by summing the numerical codes (1 or 0) assigned to the responses for corresponding questions, and coded as 1 for agree or positive response and 0 for disagree or negative response (perceived severity [max score. 4], treatment efficacy [max score. 2], self-efficacy to discuss SLD [max score. 1], and perceived susceptibility to disease risk [max score. 2]); (3) “barriers to SLD care” score was determined by summing the numbers of barriers checked on a list of 15 choices (max score. 15); and (4) “medical mistrust” score was a 1-item question regarding trust with physician judgment concerning medical care, coded as 0 for agree or positive response or 1 for disagree or negative response. The outcome measure, confidence in following provider recommendations for SLD care, was defined as sum of numerical codes: 1 for “extreme” confidence and 0 for “moderately,” “slightly,” or “not at all” responses on a Likert scale to 4 questions assessing confidence in (1) adherence to provider recommendations for SLD care; (2) weight loss; (3) alcohol cessation; and (4) family support for adhering to lifestyle modifications (max score. 4).

Descriptive analyses of patient characteristics were performed to obtain frequency (percentage) for categorical variables and median (interquartile range [IQR]) or mean (standard deviation) for continuous variables. Knowledge, belief, and barrier scores were compared using the paired t-test or chi-squared tests as appropriate. Univariate and multivariate regression modeling were performed to assess factors associated with the following outcome measures: (1) baseline knowledge; (2) change in knowledge following education; and (3) confidence to adhere to provider recommendations regarding SLD care. Predictors of interest were selected *a priori*. The multivariable models for baseline knowledge and change in knowledge was adjusted for by age, sex, race, barriers to SLD care, and baseline predictors with a *P* value < .1 using forward regression. The multivariable model for the outcome of confidence to adhere to provider recommendations was adjusted for by age, sex, race, posteducation knowledge, and baseline predictors with a *P* value < .1. For all models, race was collapsed into 4 categories as follows: non-Hispanic White, Hispanic, API, and other. The other racial category included non-Hispanic Black, mixed race, and other races due to small proportions of individuals that self-identified in these categories. As approximately 99% of participants reported trust in their physician’s judgment regarding care, the domain of mistrust was not included in the models. Moreover, English fluency and birth country were also excluded from the models due to concern of collinearity with race. Among non-White racial groups, the majority was not fluent in English (99%) and was born outside the US (96%). For all analyses, *P* < .05 was considered statistically significant. All analyses were performed using STATA 16 statistical software, Stata Corp LP, College Station, TX.

## Results

### Cohort Characteristics

Figure shows the participant flow: 401 patients were approached for SLD course education enrollment, 387 patients agreed to participate in the study, and of those, a total of 298 completed both pre-education surveys and the SLD education class while 296 participants completed a posteducation survey. Of 298 participants, cohort characteristics were as follows: median age 53 years old [IQR 43–63], 40.9% male, 55.1% Hispanic, 27.0% API, 10.5% non-Hispanic White, 2.7% non-Hispanic Black, and 4.7% other race. 63.3% had a high school education or less and 77.7% reported an annual income of less than $30,000. Median body mass index was 30.4 kg/m^2^ [IQR 27.0–35.7], 38.2% had diabetes, and 47.7% had dyslipidemia. In terms of liver disease severity, 20.1% were cirrhotic (either by biopsy or imaging). Nearly 24% of participants reported heavy alcohol use in the past year (Table 1).

**Figure:**
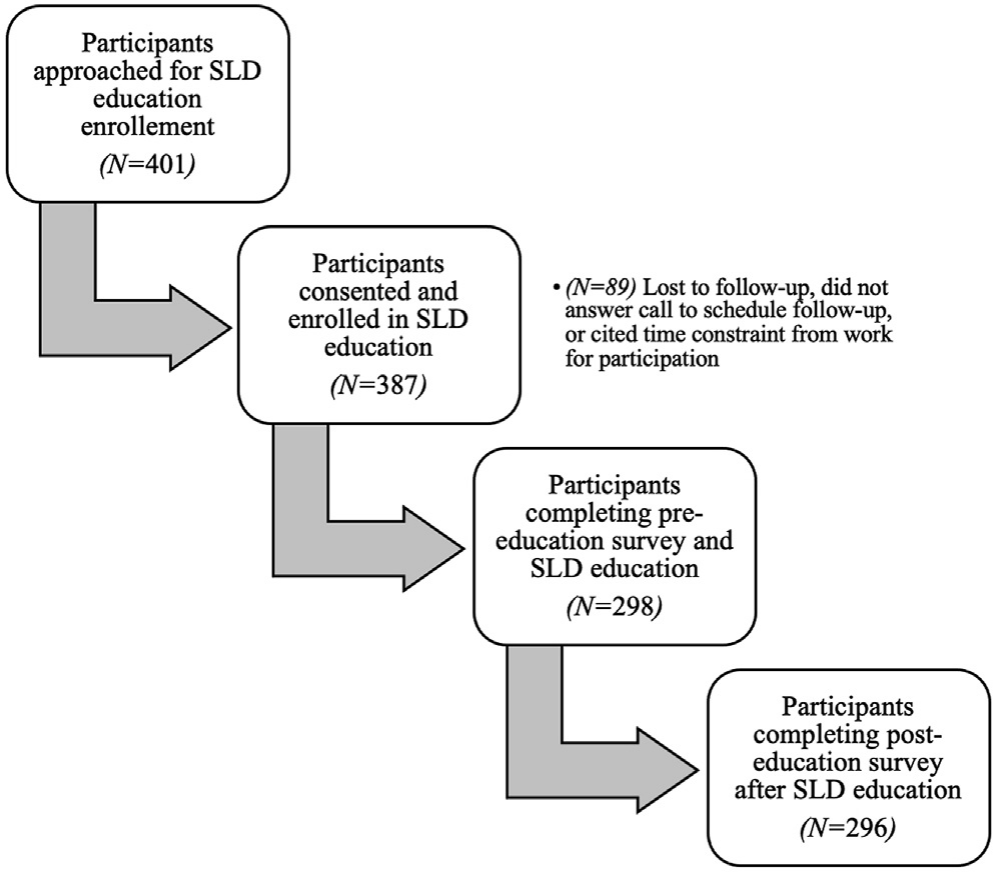
Study participant flow.

**Table 1:** Cohort Characteristics (*N* = 298)^a^

Age, median [IQR] (range), y	53 [43–63] (21–81)
Male, *N* (%)	122 (40.9%)
Race, *N* (%)	*N* = 296
White, non-Hispanic	31 (10.5%)
Black, non-Hispanic	8 (2.7%)
Hispanic	163 (55.1%)
API	80 (27.0%)
Other	14 (4.7%)
Education level, *N* (%)	*N* = 283
High school or less	179 (63.3%)
More than high school	104 (36.7%)
English fluency, *N* (%)	*N* = 291
Yes	196 (67.3%)
No	95 (32.7%)
Primary language, *N* (%)	
English	65 (21.8%)
Spanish	153 (51.3%)
Cantonese	45 (15.1%)
Other	35 (11.8%)
Birth country, *N* (%)	*N* = 291
U.S.	54 (18.5%)
Other	237 (81.4%)
Use of a translator at the doctor’s office, *N* (%)	*N* = 243
Yes	166 (68%)
No	77 (31.7%)
Alcohol use within the past year, *N* (%)	*N* = 290
None/minimal	184 (63.4%)
Moderate	37 (12.8%)
Heavy	69 (23.8%)
Reading assistance required, *N* (%)	*N* = 283
No	149 (52.7%)
Yes	134 (47.4%)
Annual income, *N* (%)	*N* = 206
<$30,000 per year	160 (77.7%)
≥$30,000 per year	46 (22.3%)
Metabolic Comorbidities, *N* (%)	
Diabetes type II	114 (38.2%)
Hyperlipidemia	142 (47.7%)
Hypertension	132 (44.3%)
Cardiovascular disease	16 (5.3%)
Chronic kidney disease	10 (3.4%)
Liver disease history, *N* (%)	
Hepatitis B	(36, 12.1%)
Hepatitis C	(11, 3.7%)
Cirrhosis by biopsy or imaging	54 out of 266 (20.3%)
Family history, *N* (%)	
Liver disease	26 (8.7%)
Hepatocellular carcinoma	7 (2.4%)
Household history of fatty liver disease, *N* (%)	*N* = 289
Yes	61 (21.1%)
No	149 (51.6%)
Do not know	79 (27.3%)
BMI (kg/m^2^), *N*, median, [IQR], (range)	*N* = 294, 30.4, [27.0–35.7], (19.4–57.4)
Laboratory tests, *N*, median, [IQR], (range)	
Alanine aminotransferase (IU/L)	*N* = 294, 48 [34–80], (12–315)
Aspartate aminotransferase (IU/L)	*N* = 293, 38 [28–60], (15–225)
Platelet count (per mcL)	*N* = 243, 241 [177–297], (39–539)
Hemoglobin A1c	*N* = 240, 6 [5.5–7.1], (3.9–13.9)
Triglycerides (mg/dl)	*N* = 239, [109–216], (32–1846)
High-dentsity lipoprotein (mg/dl)	*N* = 242, [38–56], (4–140)

BMI, body mass index.

a Unless otherwise specified in the table.

### Change in SLD Knowledge, Beliefs, Perceived Barriers to Care, and Confidence to Adhere to Provider Recommendations following Education

The mean baseline knowledge score before education was 5.8 (max. score 10) and increased to 7.7 following education (*P* < .001) (Table 2). With respect to SLD beliefs, there was an increase in prescores vs postscores across perceived severity (mean score 1.6 to 2.5, max. score 4, *P* < .001), treatment efficacy (mean score 1.0 to 1.1, max. score 2, *P* = .001), and perceived susceptibility to disease risk (mean score 1.3 to 1.8, max. score 2, *P* < .001) subdomains. Among study participants, the proportion of individuals who reported self-efficacy to discuss SLD increased by 9.4% after education. Moreover, there was a significant reduction in perceived barriers score (mean score 2.2 to 1.9, max. score 15, *P* = .003). The most cited perceived barriers to SLD care following education were as follows: “before now I was not aware that I had SLD” (33.7%), “I do not feel sick” (16.2%), “I am concerned about the cost of medical care” (16.0%), and “I fear learning more about my liver health” (15.0%). Confidence to adhere to provider recommendations also increased (mean score 2.4 to 2.7, max. score 12, *P* = .004) following education. Importantly, 98.7% of study participants reported trust in their physician’s judgment regarding their care.

**Table 2:** SLD Knowledge, Beliefs About SLD, Barriers to SLD Care, and Confidence to Adhere to Provider Recommendations Before and After SLD Education (*N* = 296)

Domains and subdomains	Preeducation score	Posteducation score	Change (in score)	*P* value
Domain 1: Knowledge [max. score 10]	5.8 ± 2.6	7.7 ± 2.0	2.0 ± 2.5	**<.001**
Domain 2: Beliefs about SLD				
(a) Perceived severity (mean ± SD) [max. score 4]	1.6 ± 1.1	2.5 ± 1.0	0.9 ± 1.3	**<.001**
(b) Treatment efficacy (mean ± SD) [max. score 2]	1.0 ± 0.5	1.1 ± 0.5	0.1 ± 0.6	**.001**
(c) Self-efficacy to discuss SLD (%) [max. score 1]	58.6	68.0	9.4	**<.001**
(d) Perceived susceptibility to disease risk (mean ± SD) [max. score 2]	1.3 ± 0.6	1.8 ± 0.5	0.5 ± 0.7	**<.001**
Domain 3: Barriers to SLD care (mean ± SD) [max. score 15]	2.2 ± 2.0	1.9 ± 1.7	−0.3 ± 2.0	**.003**
Outcome: Confidence to adhere to provider recommendations regarding SLD care (mean ± SD) [max. score 4]	2.4 ± 1.4	2.7 ± 1.4	0.3 ± 1.5	**.004**

Boldface values indicate *P* < .05.

SD, standard deviation.

### Factors Associated with Baseline SLD Knowledge

As shown in Table 3, on univariate analysis, compared to non-Hispanic White, Hispanic (coef. −1.69, 95% confidence interval (CI), −2.66 to −0.72; *P* = .001) and API (coef. −1.27, 95% CI, −2.32 to −0.21; *P* = .02) race was associated with lower baseline SLD knowledge. On the other hand, confidence to adhere to provider recommendations (coef. 0.25, 95% CI 0.05–0.46; *P* = .01) was associated with higher baseline knowledge.

**Table 3:** Univariable and Multivariable Assessment of Factors Associated With Baseline SLD Knowledge

Variables	Univariable analysis				Multivariable analysis (*N* = 279)		
	*N*	Coef.	95% CI	*P* value	Coef.	95% CI	*P* value
Age (in decades)	*N* = 296	−0.06	−0.29 to 0.17	.60	−0.01	−0.19 to 0.17	.92
Female sex, ref male	*N* = 296	−0.18	−0.78 to 0.41	.54	0.03	−0.45 to 0.51	.91
Race	*N* = 298						
White, non-Hispanic		Ref	–	–	–	–	–
Hispanic		**−1.69**	**−2.66 to −0.72**	**.001**	−0.67	−1.57 to 0.23	.14
API		**−1.27**	**−2.32 to −0.21**	**.02**	−0.12	−1.02 to 0.77	.79
Other		−1.25	−2.99 to 0.49	.10	−0.86	−2.27 to 0.55	.23
Education level	*N* = 285						
High school or less		Ref	–	–	–	–	–
More than high school		**0.82**	**−0.21 to 1.43**	**.01**	**0.62**	**0.08**–**1.15**	**.03**
Annual income	*N* = 207						
<$30,000 per year		Ref	–	–	–	–	–
≥$30,000 per year		0.72	−0.14 to 1.57	.10	–	–	–
Alcohol use within the past year	*N* = 293						
None/minimal		Ref	–	–	–	–	–
Moderate		0.21	−0.68 to 1.10	.64	–	–	–
Heavy		−0.41	−1.13 to 0.31	.27	–	–	–
Beliefs about SLD	*N* = 298						
(a) Perceived everity		**1.20**	**0.98−1.42**	**<.001**	**0.62**	**0.37**–**0.65**	**<.001**
(b) Treatment efficacy		**2.34**	**1.87−2.81**	**<.001**	**1.38**	**0.93−1.83**	**<.001**
(c) Self-efficacy to discuss SLD		**1.83**	**1.28−2.38**	**<.001**	**0.71**	**0.21−1.21**	**.006**
(d) Perceived susceptibility to disease risk		**1.85**	**1.44−2.27**	**<.001**	**0.93**	**0.54−1.33**	**<.001**
Barriers to SLD Care	*N* = 298	−0.10	−0.24 to 0.05	.20	−0.05	−0.16 to 0.06	.36
Confidence to adhere to provider recommendations regarding SLD Care	*N* = 298	**0.25**	**0.05**−**0.46**	**.01**	0.02	−0.14 to 0.19	.79

Boldface values indicate *P* < .05.

On multivariate models, adjusted for age, sex, race, and barriers to SLD care, greater than high school education (vs high school or less) was associated with higher baseline knowledge scores (coef. 0.62, 95% CI, 0.08–1.15; *P* = .03). Beliefs about SLD were also associated with baseline knowledge (Table 3).

### Factors Associated with Change in SLD Knowledge

On univariate analysis, baseline beliefs (perceived severity, treatment efficacy, self-efficacy to discuss SLD, perceived susceptibility to disease) and baseline confidence to adhere to provider recommendations were associated with lesser change in knowledge following education (Table 4).

**Table 4:** Univariable and Multivariable Analysis of Factors Associated With Change in SLD Knowledge Following Education

Variables	Univariable analysis				Multivariable analysis (*N* = 285)		
	*N*	Coef.	95% CI	*P* value	Coef.	95% CI	*P* value
Age (in decades)	*N* = 291	−0.02	−0.24 to 0.21	.89	0.01	−0.20 to 0.22	.92
Female sex, ref male	*N* = 291	0.04	−0.54 to 0.61	.90	0.29	−0.27 to 0.85	.31
Race	*N* = 295						
White, non-Hispanic		Ref	–	–	–	–	–
Hispanic		−0.17	−1.12 to 0.79	.73	−0.71	−1.67 to 0.24	.14
API		−0.31	−1.35 to 0.73	.56	−0.75	−1.72 to 0.21	.13
Other		−0.28	−1.93 to 1.38	.74	−0.41	−1.94 to 1.13	.60
Education level	*N* = 282						
High school or less		Ref	–	–	–	–	–
More than high school		0.40	−0.18 to 0.97	.18	–	–	–
Annual income	*N* = 204						
<$30,000 per year		Ref	–	–	–	–	–
≥$30,000 per year		0.02	−0.86 to 0.89	.97	–	–	–
Alcohol use within the past year	*N* = 289						
None		Ref	–	–	–	–	–
Minimal/moderate		0.26	−0.58 to 1.10	.55	0.06	−0.72 to 0.85	.87
Heavy		**0.88**	**0.18−1.58**	**.01**	**0.74**	**0.07−1.40**	**.03**
Baseline beliefs about SLD	*N* = 296						
(a) Perceived severity		**−0.87**	**−1.10 to −0.64**	**<.001**	**−0.52**	**−0.79 to −0.24**	**<.001**
(b) Treatment efficacy		**−1.50**	**−1.99 to −1.01**	**<.001**	**−0.72**	**−1.25 to −0.19**	**.008**
(c) Self-efficacy to discuss SLD		**−1.12**	**−1.69 to −0.56**	**<.001**	−0.54	−1.12 to 0.05	.07
(d) Perceived susceptibility to disease risk		**−1.14**	**−1.57 to −0.71**	**<.001**	−0.44	−0.92 to 0.04	.07
Baseline barriers to SLD Care	*N* = 296	0.11	−0.03 to 0.25	.127	0.07	−0.06 to 0.20	.28
Baseline Confidence to adhere to provider recommendations regarding SLD Care	*N* = 245	**−0.33**	**−0.52 to −0.14**	**.001**	−0.11	−0.31 to 0.08	.25

Boldface values indicate *P* < .05.

On multivariate analysis, interestingly, heavy alcohol use in the last year (vs none) (coef. 0.74, 95% 0.07–1.40; *P* = .03) was associated with greater change in knowledge following education. Perceived severity (coef. −0.52, 95% CI, −0.79 to −0.24; *P* < .001) and treatment efficacy (coef. −0.72, 95% CI, −1.25 to −0.19; *P* = .008) were associated with lesser change in knowledge scores after education (Table 4).

### Factors Associated with Confidence to Follow Provider Recommendations after Education

On univariate analysis, factors associated with greater confidence to adhere to provider recommendations following SLD education included female sex (coef. 0.44, 95% CI, 0.12–0.76; *P* = .008), perceived severity (coef. 0.17, 95% CI, 0.02–0.33; *P* = .03), treatment efficacy (coef. 0.45, 95% CI, 0.21–0.68; *P* < .001), and self-efficacy to discuss SLD (coef. 0.40, 95% CI, 0.06–0.74; *P* = .02) (Table 5). Conversely, heavy alcohol use in the last year (vs none) (coef. −0.48, 95% CI, −0.88 to −0.07; *P* = .02) and posteducation barriers to SLD (coef. −0.16, 95% CI, −0.25 to −0.06; *P* = .001) were associated with lower confidence to adhere to provider recommendations following education.

**Table 5:** Factors Associated With Confidence to Adhere to Provider Recommendations Regarding SLD Care following Education

Variables	Univariable analysis (*N* = 298)			Multivariable analysis (*N* = 287)		
	Coef.	95% CI	*P* value	Coef.	95% CI	*P* value
Age (in decades)	0.10	−0.02 to 0.23	.12	**0.18**	**0.04**−**0.31**	**.01**
Female sex, ref male	**0.44**	**0.12**−**0.76**	**.008**	0.26	−0.08 to 0.60	.13
Race						
White, non-Hispanic	Ref	–	–	–	–	–
Hispanic	**0.70**	**0.17**−**1.23**	**.01**	**0.64**	**−0.03 to 1.25**	**.04**
API	0.14	−0.44 to 0.71	.64	0.03	−0.59–0.65	.92
Other	**1.38**	**0.47 to 2.29**	**.003**	**1.26**	**0.28**−**2.24**	**.01**
Education level						
High school or less	Ref	–	–	–	–	–
More than high school	−0.33	−0.67 to 0.01	.05	−0.25	−0.63 to 0.12	.19
Annual income						
<$30,000 per year	Ref	–	–	–	–	–
≥$30,000 per year	−0.10	−0.58 to 0.38	.68	–	–	–
Alcohol use within the past year						
None/minimal	Ref	–	–	–	–	–
Moderate	−0.30	−0.79 to 0.18	.20	−0.09	−0.58 to 0.39	.71
Heavy	**−0.48**	**−0.88 to −0.07**	**.02**	−0.36	−0.77 to 0.05	.09
Posteducation knowledge	0.01	−0.06–0.09	.71	−0.02	−0.12 to 0.08	.73
Posteducation beliefs about SLD						
(a) Perceived everity	**0.17**	**0.02−0.33**	**.03**	0.17	−0.02 to 0.36	.09
(b) Treatment efficacy	**0.45**	**0.21−0.68**	**<.001**	0.05	−0.32 to 0.42	.80
(c) Self-efficacy to discuss SLD	**0.40**	**0.06−0.74**	**.02**	**0.38**	**0.01−0.74**	**.04**
(d) Perceived susceptibility to disease risk	0.24	−0.06 to 0.54	.11	–	–	–
Posteducation barriers to SLD Care	**−0.16**	**−0.25 to −0.06**	**.001**	**−0.14**	**−0.24 to −0.04**	**.008**

Boldface values indicate *P* < .05.

On multivariate analysis, compared to non-Hispanic White, Hispanic (coef. 0.64, 95% CI, −0.03 to 1.25; *P* = .04) and other race (coef. 1.26, 95% CI, 0.28–2.24; *P* = .01) was associated with higher confidence to adhere to provider recommendations following education. Older age (coef. 0.17, 95% CI, 0.03–0.30; *P* = .014) and self-efficacy to discuss SLD (coef. 0.38, 95% CI 0.01–0.74; *P* = .04) was also associated with greater confidence to adhere to provider recommendations while perceived barriers to SLD care (coef. −0.14, 95% CI, −0.24 to −0.04; *P* = .008) was associated with lower confidence levels after education (Table 5).

## Discussion

In this study, we tested the effect of a formal SLD educational intervention in a diverse, safety net population. Our intervention improved scores across all domains of knowledge, beliefs about SLD, and barriers to SLD care as well as confidence to adhere to provider recommendations. Interestingly, those who reported heavy alcohol use, a group especially vulnerable to SLD, were particularly receptive to this intervention in changing their level of knowledge. Moreover, Hispanic individuals attained higher confidence to adhere to provider recommendations regarding SLD care following education.

Previous studies have identified significant gaps in patient knowledge of SLD, particularly regarding the natural history of the disease.^3^^,^^23^ Glass et al. further assessed the impact of an educational intervention, demonstrating improvement in knowledge levels and clinical outcomes related to SLD. While this study adds to literature in educational interventions for SLD, it was limited by lack of racial diversity as 85% in their cohort identified as White. Our study focuses on vulnerable populations known to be disproportionately affected by SLD with 90% of participants identified as non-White and the majority was born outside of the US with limited English fluency, which may be more representative of the contemporary SLD population in the US.

Although the determinants of disease knowledge attainment are complex and multifactorial, access to care is often limited in racial groups due to social risk factors, emphasizing the importance of testing SLD interventions among diverse and vulnerable populations. Importantly, on univariate analysis, we found that Hispanic, API, and other races were associated with lower baseline SLD knowledge. These findings likely reflect underlying structural and social barriers to hepatology care, such as, cultural differences, language discordance, and underinsurance/uninsurance as well as provider discomfort with discussing SLD care.^13^^,^^14^^,^^24^ However, when accounting for race among other factors, our analysis demonstrated that attitudes and beliefs regarding SLD and education level rather than racial identity or perceived barriers to SLD care were the significant drivers of baseline SLD knowledge.

The Transtheoretical (Stages of Change) Model emphasizes that disease-related knowledge is especially important to propel patients from the precontemplative to contemplative stage of change, eventually leading to action and maintenance of healthy behaviors. While our educational program improved overall patient knowledge, we found this intervention to be particularly beneficial among those with heavy alcohol use. As the prevalence of combined metabolic dysfunction and alcohol-associated liver disease (MetALD) is higher than previously estimated in the US population at approximately 2%,^1^ we now recognize that alcohol reduction/cessation along with cardiometabolic optimization (eg, healthy eating, physical activity, and weight loss) as critical to the management of these individuals.^1^^,^^25^, ^26^, ^27^ Furthermore, obesity and other components of metabolic syndrome are major risk factors for the development of advanced liver disease.^28^ These recent findings highlight the novelty of our intervention in furthering patient knowledge regarding alcohol reduction/cessation along with adoption of other healthy lifestyle behaviors as an important component of SLD care. Future studies should assess the impact of formal SLD education in curbing unhealthy alcohol use and promoting uptake of healthy lifestyle behaviors on clinical outcomes, especially among vulnerable populations.

Finally, we found that Hispanic individuals, even after controlling for barriers to care, were particularly receptive to an educational program in improving confidence to adhere to provider recommendations regarding SLD care. This observation is timely as SLD disproportionately affects Hispanic individuals and thus patient-centered educational programs are simple interventions that reduce health disparities in this population. In fact, across all subtypes of SLD (MASLD, MASLD-predominant MetALD, associated liver disease–predominant MetALD, and associated liver disease), prevalence of disease is now the highest in Hispanic adults.^2^ Moreover, social and structural barriers limit access to hepatology care in this vulnerable population, leading to significant liver-related morbidity and mortality.^29^^,^^30^ Other groups especially amenable to this educational intervention leading to an improvement in confidence to adhere to provider recommendations included elderly individuals. Although this remains unexplored in our study cohort, we suspect older patients also scored highest in health care utilization. In fact, older individuals visit health care providers twice as much as those under the age of 65.^31^ More interface with the health-care system likely opens up further opportunities to discuss and/or reinforce SLD topics with nonhepatology providers, potentially improving confidence to follow provider recommendations. Additional tailored interventions, in conjunction with education, may be necessary to enhance confidence to adhere to SLD care among younger populations.

While self-efficacy to discuss also played a role, as expected, perceived barriers to SLD care were independently associated with lower confidence to adhere to provider recommendations regarding lifestyle modification. Thus, provider awareness and focus on addressing patient-reported barriers to SLD care will likely play a significant role in enhancing patient confidence and success to adhere to provider recommendations. We have previously shown that patient-reported barriers to lifestyle modification in SLD included knowledge gaps, perceptions of family practices affecting personal behaviors, self-reported anxiety on health behaviors, and physical or logistical limitations.^32^ A recent scoping review also highlighted additional barriers including lack of social opportunity (eg, cultural influences, lack of family support, responsibilities at home, and work schedules).^33^ Therefore, routine screening for modifiable barriers by health-care providers, including social needs and referral to community-based resources, represents a potential strategy to promote uptake of lifestyle modification.^34^

Our study has several limitations. Self-report, recall bias, and response bias are inherent to any survey-based study. The relatively small sample size of our study at a safety net health system among patients engaged in liver specialty care limits generalizability across other settings. Data on receipt of in-person vs virtual education was not captured. Nevertheless, this study represents the largest reported assessment of knowledge attainment in this population to date. In addition, we report on postsurvey data that was conducted immediately after the educational intervention and the long-term clinical outcomes of patients following education is not known. Further work examining long-term retention of knowledge and changes in attitudes and barriers as well as clinical outcomes following education is needed and is currently in progress.

In summary, our study demonstrated the efficacy of a formal SLD education program in a diverse and vulnerable patient population. We observed positive change in knowledge and confidence to follow provider recommendations on lifestyle modification following education among at-risk groups, particularly those with heavy alcohol use, older patients, and Hispanics. Indeed, we demonstrated that a formal and patient-centered educational intervention is effective in empowering at-risk populations to adhere to recommended treatment plans and represents an integral component of SLD management and care for safety net populations.

## Supplementary Materials

Table A1

## References

[1] Lee B.P., Dodge J.L., Terrault N.A. (2024). National prevalence estimates for steatotic liver disease and subclassifications using consensus nomenclature. Hepatology.

[2] Ochoa-Allemant P., Marrero J.A., Serper M. (2023). Racial and ethnic differences and the role of unfavorable social determinants of health across steatotic liver disease subtypes in the United States. Hepatol Commun.

[3] Tincopa M.A., Wong J., Fetters M. (2021). Patient disease knowledge, attitudes and behaviours related to non-alcoholic fatty liver disease: a qualitative study. BMJ Open Gastroenterol.

[4] Singh A., Dhaliwal A.S., Singh S. (2020). Awareness of nonalcoholic fatty liver disease is increasing but remains very low in a representative US cohort. Dig Dis Sci.

[5] Cleveland E.R., Ning H., Vos M.B. (2019). Low awareness of nonalcoholic fatty liver disease in a population-based cohort sample: the CARDIA study. J Gen Intern Med.

[6] Younossi Z.M., Ong J.P., Takahashi H. (2022). A global survey of physicians knowledge about nonalcoholic fatty liver disease. Clin Gastroenterol Hepatol.

[7] Rinella M.E., Neuschwander-Tetri B.A., Siddiqui M.S. (2023). AASLD practice guidance on the clinical assessment and management of nonalcoholic fatty liver disease. Hepatology.

[8] Crabb D.W., Im G.Y., Szabo G. (2020). Diagnosis and treatment of alcohol-associated liver diseases: 2019 practice guidance from the American Association for the Study of Liver Diseases. Hepatology.

[9] Lubega S., Agbim U., Surjadi M. (2013). Formal hepatitis C education enhances HCV care coordination, expedites HCV treatment and improves antiviral response. Liver Int.

[10] Surjadi M., Torruellas C., Ayala C. (2011). Formal patient education improves patient knowledge of hepatitis C in vulnerable populations. Dig Dis Sci.

[11] Mukhtar N.A., Evon D.M., Yim C. (2021). Patient knowledge, beliefs and barriers to hepatitis B care: results of a multicenter, multiethnic patient survey. Dig Dis Sci.

[12] Khalili M., Powell J., Park H.H. (2022). Shelter-based integrated model is effective in scaling up hepatitis C testing and treatment in persons experiencing homelessness. Hepatol Commun.

[13] National Research Council (US) Panel on Race/Ethnicity (2004). Behavioral health interventions: What works and why?.

[14] Bastani R., Glenn B.A., Taylor V.M. (2010). Integrating theory into community interventions to reduce liver cancer disparities: the Health Behavior Framework. Prev Med.

[15] Partida D., Powell J., Ricco M. (2022). Formal hepatitis C education increases willingness to receive therapy in an on-site shelter-based HCV model of care in persons experiencing homelessness. Open Forum Infect Dis.

[16] Alvidrez J., Castille D., Laude-Sharp M. (2019). The national institute on minority health and health disparities research framework. Am J Public Health.

[17] Maxwell A.E., Bastani R., Glenn B.A. (2014). Developing theoretically based and culturally appropriate interventions to promote hepatitis B testing in 4 Asian American populations, 2006-2011. Prev Chronic Dis.

[18] Egede L.E., Ellis C. (2008). Development and testing of the multidimensional trust in health care systems scale. J Gen Intern Med.

[19] Thompson H.S., Valdimarsdottir H.B., Winkel G. (2004). The Group-Based Medical Mistrust Scale: psychometric properties and association with breast cancer screening. Prev Med.

[20] National Institute on Alcohol Abuse and Alcoholism Alcohol consumption questionnaire. https://www.niaaa.nih.gov/research/nesarc-iii/questionnaire.

[21] Rinella M.E., Lazarus J.V., Ratziu V. (2023). A multisociety Delphi consensus statement on new fatty liver disease nomenclature. Hepatology.

[22] WHO Expert Consultation (2004). Appropriate body-mass index for Asian populations and its implications for policy and intervention strategies. Lancet.

[23] Glass L., Asefa H., Volk M. (2022). Disease knowledge, health-related quality of life, and lifestyle behavior change in patients with nonalcoholic fatty liver disease: impact of an educational intervention. Dig Dis Sci.

[24] Morrill K.E., Crocker R.M., Hingle M.D. (2021). Awareness, knowledge, and misperceptions related to nonalcoholic fatty liver disease in a community sample of Mexican-origin women: a mixed methods study. Front Public Health.

[25] Kalligeros M., Vassilopoulos A., Vassilopoulos S. (2024). Prevalence of steatotic liver disease (MASLD, MetALD, and ALD) in the United States: NHANES 2017-2020. Clin Gastroenterol Hepatol.

[26] Yeo Y.H., Zhu Y., Arab J.P. (2023). Alcohol intake thresholds among individuals with steatotic liver disease. JAMA Netw Open.

[27] Long M.T., Massaro J.M., Hoffmann U. (2020). Alcohol use is associated with hepatic steatosis among persons with presumed nonalcoholic fatty liver disease. Clin Gastroenterol Hepatol.

[28] Chiang D.J., McCullough A.J. (2014). The impact of obesity and metabolic syndrome on alcoholic liver disease. Clin Liver Dis.

[29] Jones P.D., Lai J.C., Bajaj J.S. (2023). Actionable solutions to achieve health equity in chronic liver disease. Clin Gastroenterol Hepatol.

[30] Kardashian A., Serper M., Terrault N. (2023). Health disparities in chronic liver disease. Hepatology.

[31] Institute of Medicine (US) Committee on the Future Health Care Workforce For older Americans (2008).

[32] Medina S.P., Kim R.G., Magee C. (2023). Cross-sectional study on stigma and motivation to adhere to lifestyle modification among vulnerable populations with fatty liver disease. Obes Sci Pract.

[33] Shibayama K., Furushima C., Saka M. (2024). Barriers to lifestyle modification in patients with non-alcoholic fatty liver disease: a scoping review. J Rural Med.

[34] Hacker K., Auerbach J., Ikeda R. (2022). Social determinants of health-an approach taken at CDC. J Public Health Manag Pract.

