# Supplementary material for: Steatotic Liver Disease Education Enhances Knowledge and Confidence to Adhere to Provider Recommendations in Diverse and Vulnerable Populations
Source: Gastro Hep Adv. 2024 Nov 20;4(3):100589. doi: 10.1016/j.gastha.2024.11.005 (PMC11808611; doi:10.1016/j.gastha.2024.11.005)
Supplement: Table A1 [file mmc1.docx]

**Supplemental Table 1**. Fatty Liver Disease Questionnaire: Knowledge, Attitude, and Barriers to Care

| **Domain 1. Knowledge** | | | | | |
| --- | --- | --- | --- | --- | --- |
| Survey Question | Responses (N, (%)) | | | | |
|  | Yes | No | I don’t know |  |  |
| 1. Fatty liver can cause cirrhosis. |  |  |  |  |  |
| 1. It is possible to have more than one type of liver disease (for example, fatty liver disease and viral hepatitis). |  |  |  |  |  |
| 1. It is okay for people with fatty liver to keep drinking alcohol. |  |  |  |  |  |
| 1. Fatty liver disease is very rare. |  |  |  |  |  |
| 1. Fatty liver can be treated with diet and exercise. |  |  |  |  |  |
| 1. Diabetes is not a risk factor for fatty liver disease. |  |  |  |  |  |
| 1. If people with fatty liver lose weight, their fatty liver disease may get better. |  |  |  |  |  |
| 1. High cholesterol affects my heart but not my liver. |  |  |  |  |  |
| 1. People with fatty liver disease may have no symptoms. |  |  |  |  |  |
| 1. Drinking alcohol (for example beer, wine, hard liquor) increases a person’s risk for fatty liver. |  |  |  |  |  |
| **Domain 2: Beliefs about FLD** | | | | | |
| **2a. Perceived severity** | | | | | |
| Survey Question | Responses (N, (%)) | | | | |
|  | Yes | No | I don’t know |  |  |
| 1. How concerned are you about your fatty liver? |  |  |  |  |  |
| 1. Some persons can die from fatty liver disease. |  |  |  |  |  |
| 1. If someone has liver scarring from fatty liver disease, this can lead to liver cancer. |  |  |  |  |  |
| 1. Fatty liver can rarely cause heart problems. |  |  |  |  |  |
| **2b. Treatment efficacy** | | | | | |
| Survey Question | Responses (N, (%)) | | | | |
|  | Yes/True | No/False | I don’t know |  |  |
| 1. Fatty liver can be treated with medications |  |  |  |  |  |
| 1. Weight loss and exercise are the most important things someone can do to prevent fatty liver disease from getting worse. |  |  |  |  |  |
| **2c. Self-efficacy to discuss FLD** | | | | | |
| Survey Question | Responses (N, (%)) | | | | |
|  | Yes/True | No/False | I don’t know |  |  |
| 1. Have you ever discussed your liver problem with people you know such as your family or friends? |  |  |  |  |  |
|  |  |  |  |  |  |
| **2d. Perceived susceptibility to disease risk** | | | | | |
| Survey Question | Responses (N, (%)) | | | | |
| 1. People with fatty liver disease are at risk of heart disease | Yes/True | No/False | I don’t know |  |  |
| 1. Do you think you have a liver problem? |  |  |  |  |  |
| **Domain 3: Barriers** | | | | | |
| What barriers (if any) do you experience in seeking care for or managing your fatty liver disease? | | | | | |
| **Domain 4: Medical Mistrust** | | | | | |
| Survey Question | Responses (N, (%)) | | | | |
|  | Yes | No | Decline to answer |  |  |
| 1. I am going to ask you a question about your thoughts on medical research and medical care. Please let us know if you agree or disagree with the following statement: I can trust my doctor’s judgments concerning my medical care. |  |  |  |  |  |
| **Outcome: Confidence in Following Provider Recommendations for SLD Care** | | | | | |
| Survey Question | Responses (N, (%)) | | | | |
| How confident are you that you can… | Not at all | Slightly | Moderately | Extremely | Decline to answer |
| 1. Follow your provider’s recommendations for management of your liver problem. |  |  |  |  |  |
| 1. Lose weight |  |  |  |  |  |
| 1. Stop any alcohol use |  |  |  |  |  |
| 1. Count on your family for support in following your provider’s recommendations for diet and exercise. |  |  |  |  |  |
